# Semantic Segmentation of Building Roof in Dense Urban Environment with Deep Convolutional Neural Network: A Case Study Using GF2 VHR Imagery in China

**DOI:** 10.3390/s19051164

**Published:** 2019-03-07

**Authors:** Yuchu Qin, Yunchao Wu, Bin Li, Shuai Gao, Miao Liu, Yulin Zhan

**Affiliations:** 1 State Key Lab of Remote Sensing Sciences, Institute of Remote Sensing and Digital Earth (RADI), Chinese Academy of Sciences (CAS), Beijing 100101, China; libin01@radi.ac.cn (B.L.); gaoshuai@radi.ac.cn (S.G.); zhanyl@radi.ac.cn (Y.Z.); 2 Beijing Municipal Institute of City Planning & Design, Beijing 100045, China; ycwu66@gmail.com; 3 Department of Mathematics & Statistics, South Dakota State University, Brookings, 57006 SD, USA; loveliumiao@hotmail.com

**Keywords:** VHR image, building roof, segmentation, GF2, deep convolution neural network

## Abstract

This paper presents a novel approach for semantic segmentation of building roofs in dense urban environments with a Deep Convolution Neural Network (DCNN) using Chinese Very High Resolution (VHR) satellite (i.e., GF2) imagery. To provide an operational end-to-end approach for accurately mapping build roofs with feature extraction and image segmentation, a fully convolutional DCNN with both convolutional and deconvolutional layers is designed to perform building roof segmentation. We selected typical cities with dense and diverse urban environments in different metropolitan regions of China as study areas, and sample images were collected over cities. High performance GPU-mounted workstations are employed to perform the model training and optimization. With the building roof samples collected over different cities, the predictive model with convolution layers is developed for building roof segmentation. The validation shows that the overall accuracy (OA) and the mean Intersection Over Union (mIOU) of DCNN-based semantic segmentation results are 94.67% and 0.85, respectively, and the CRF-refined segmentation results achieved OA of 94.69% and mIOU of 0.83. The results suggest that the proposed approach is a promising solution for building roof mapping with VHR images over large areas in dense urban environments with different building patterns. With the operational acquisition of GF2 VHR imagery, it is expected to develop an automated pipeline of operational built-up area monitoring, and the timely update of building roof map could be applied in urban management and assessment of human settlement-related sustainable development goals over large areas.

## 1. Introduction

Urbanization is a process whereby human beings significantly affect the natural environment of land surfaces. It not only causes changes in land cover and land use, but also has profound effects on the daily life of our society [1]. Buildings are the essential part of urban environment. They play a vital role in human life as a basic infrastructure of human settlement [2]. Accurate and timely maps of buildings are crucial for urban planning, environmental management and sustainable development studies, especially in areas undergoing rapid urbanization [3,4]. However, the conventional ways of building roof maps, e.g., field surveys or manual annotation of imagery, are time-consuming and labor-intensive endeavors. The widely available Very High Resolution (VHR) satellite imagery is an unique data source for building roof mapping [5,6]. Many studies were carried out to extract building roofs. However, most of the methods are based on specific features and rules, and low-level features were designed for mapping building with VHR images, e.g., histogram of oriented gradients (HOG) [7,8]. In addition, some other studies for building detection also tried use shadow information [9,10], graph theory [11,12], or a MRF-based approach [13]. The classification methods take advantage of multispectral information [14,15]. Huang et al. proposed a post-processing framework for building roof classification using VHR imagery, where the framework relies on a morphological building index (MBI). It integrated spectral, geometrical, and contextual information for building mapping, and experiments suggested that the proposed framework achieved good results [16]. However, these methods rely on the selected features and image types, it is hard to develop a model for operational building mapping over large areas, especially for dense urban environments with different building types. 

As an important machine learning approach, Neural Networks (NNs) are inspired by the process of the recognition process of brains to perform recognition tasks. Since 2006, a series of techniques and strategies, e.g., layer-wise training and pre-training, Restricted Boltzmann Machine (RBM), Recurrent Neural Networks (RNNs) were proposed for NNs with multiple hidden layers for large scale learning problems [17]. Deep NNs with multiple hidden layers are applied for learning data representations in recognition tasks, e.g., image classification, scene understanding, speech recognition, etc. Convolutional Neural Network (CNNs) are designed for feature learning as well as inference in image classification, segmentation and scene understanding [18]. CNNs take a moving window as a filter for capturing features in image space, and the features then can be applied for classification or segmentation [19]. CNNs could reduce the number of weights and bias in NNs by sharing deep neural network parameters, which provide a solution for pixel level recognition with a reasonable model size. CNN is also a promising way to combine features from both the spectral and spatial textural domains for scene understanding and segmentation. Currently, with high performance GPUs, CNNs have been widely applied in vision recognition tasks, and have achieved state-of the-art results [18,20,21,22,23]. There are many efforts for building mapping with DCNNs. Yuan proposed automatic building extraction methods in aerial scenes using convolutional neural networks [24]. A network was designed with novel components which are easy to implement, which enables the network to learn hierarchical features for segmenting buildings. Bittner et al. proposed a method which automatically generates a full resolution binary building mask using a Fully Convolution Network (FCN) architecture and achieved promising results for building roof mapping [25]. Sun et al., developed a two-stage CNN model to detect rural buildings in high-resolution imagery [26]. The experiments showed that the two-stage CNN model reduced the complexity of the background information and improved the efficiency, achieving an overall accuracy of 88%. Besides these methods, the building detection task can also be solved as part of the land use classification problem with deep learning methods. For example, Maggiori et al. investigated convolutional neural networks for large-scale remotely sensed image classification, proposing an end-to-end framework for the dense, pixel-wise classification of satellite imagery with CNNs [27]. Microsoft developed a building footprint data set for the USA using Bing Map images with DCNN [28]. To promote the state-of-the-art results in satellite image analysis, the DeepGlobe Satellite Challenge was hosted by Facebook, DigitalGlobe etc. Commercial VHR images and associated ground truth were provided by DigitalGlobe for road extraction, land cover classification and building detection, and it provided state-of-the-art algorithms for satellite image analysis [29]. 

So far, it has been well addressed that the high resolution satellite could capture VHR imagery that covers large areas in a timely way with pre-trained CNN models. It is feasible to conduct large built-up area mapping at an individual building level. However, operational mapping with VHR imagery over large areas is still a costly task with current commercial VHR images. In addition, we don’t have low cost or free data sets for developing countries with dense and diverse urban environments. To support urban planning and management, and sustainable development assessment over large areas, especially to provide timely and accurate building information for developing countries, affordable or free VHR images like GF2 PMS imagery would advance the remote sensing technology to broad domains in SDGs assessment. Gaofen-2 (GF2) is one of the series satellite missions in China’s High-resolution Earth Observation System (CHEOS) [30]. It was launched on August 19, 2014 with a panchromatic and multispectral sensor (PMS) which acquires panchromatic and multispectral images simultaneously. It is expected to provide a long-term VHR satellite imagery at an affordable cost with global coverage, especially of developing countries. 

This study aims to examine the potential of the GF2 PMS for building roof mapping over large urban areas, especially in dense and diverse urban environments. It presents our efforts at building roof segmentation with DCNN over mega-cities in China, using a DCNN model. With the development of an automatic pipeline for operational building mapping with Chinese GF2 VHR imagery over large areas, regardless of the location of the areas and the acquisition data of the image it is expected to provide an alternative solution for supporting sustainable development assessment and urban planning with VHR imagery at low cost.

## 2. Materials and Methods

### 2.1. Data Sets and Study Areas

The GF2 PMS is capable of collecting VHR imagery with a Ground Sampling Distance (GSD) of of 0.8 m panchromatic and 3.2 m multispectral bands on a swath of 45 km. Table 1 shows the basic configurations of the sensor.

To cover the diverse urban patterns and building styles in China, typical cities across the country were selected in different metropolitan regions as study areas, and training and test GF2 PMS images were collected with manual delineation. Table 2 lists the cities and acquisition date of the images. With the assumption that imagery acquired during the growing season would provide better visual effects in both the spectral and spatial domains, we only choose images acquired in the period between June and September in 2016 with cloud cover of less than 5%, and a total of seven images were selected in this study for further processing.

#### 2.1.1. Image Preprocessing and Training Data Collection

**Figure 1:**
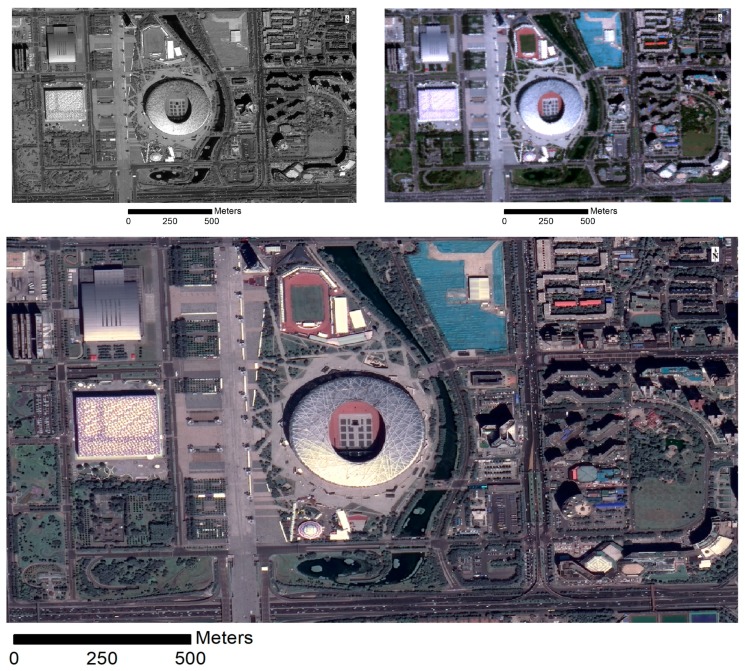
Sample images captured by GF2 PMS sensors at 27 August 2016: the three images cover the same area around the National Stadium at Beijing, where the upper left is a panchromatic image with resolution of 1.0 m, the upper right is the true color composite of a multi-spectral image with resolution of 4.0 m, and the bottom one is the pan-sharped image of the panchromatic and multi-spectral images.

Though the orientation of PMS images is well calibrated, high precision co-registration between the panchromatic and multispectral images acquired by PMS is performed to keep rigorous geometrical alignment. With the reference of corresponding panchromatic imagery, visual selection of ground control points is carried out for georectification of the multispectral images. The overall mean registration error for all images is less than 1.0 m. With the panchromatic and the corresponding rectified multispectral image, pan-sharping is conducted to obtain fused images with spatial resolution of 1.0 m using a Gram-Schmidt algorithm. Figure 1 illustrates the PMS panchromatic image, the true color composites of PMS multispectral image, and the true color composites of pan-sharped images. The images shows that the pan-sharping could enhance the characteristics of the building boundaries with much higher resolution.

#### 2.1.2. Collection of Sample Images

With the pan-sharped high resolution imagery, sample data set was collected by manual delineation, though the basic unit of delineation is individual building, for those closely connected buildings, neighbour buildings are merged together since it is hard to separate individual buildings. And the manual delineated polygons are then converted to binary images as building roof mask, i.e., the binary image values of 1 and 0 means building roof, rest of land cover types respectively. 

### 2.2. Methodology

In this paper, a DCNN model is developed to perform the feature extraction and per-pixel labeling for building roof upon the VHR satellite imagery. Generally, a DCNN model consists of convolutional and pooling layers, where the convolutional layers perform feature extraction and the pooling layers summarize the features by aggregating neighbour pixels in imagery, while both convolutional and pooling layers reduce image size, the deconvolutional layer provide a way to upsample image to original resolution, the detailed description of the three type operations is well introduced in [31].

#### 2.2.1. Design of DCNN

**Figure 2:**
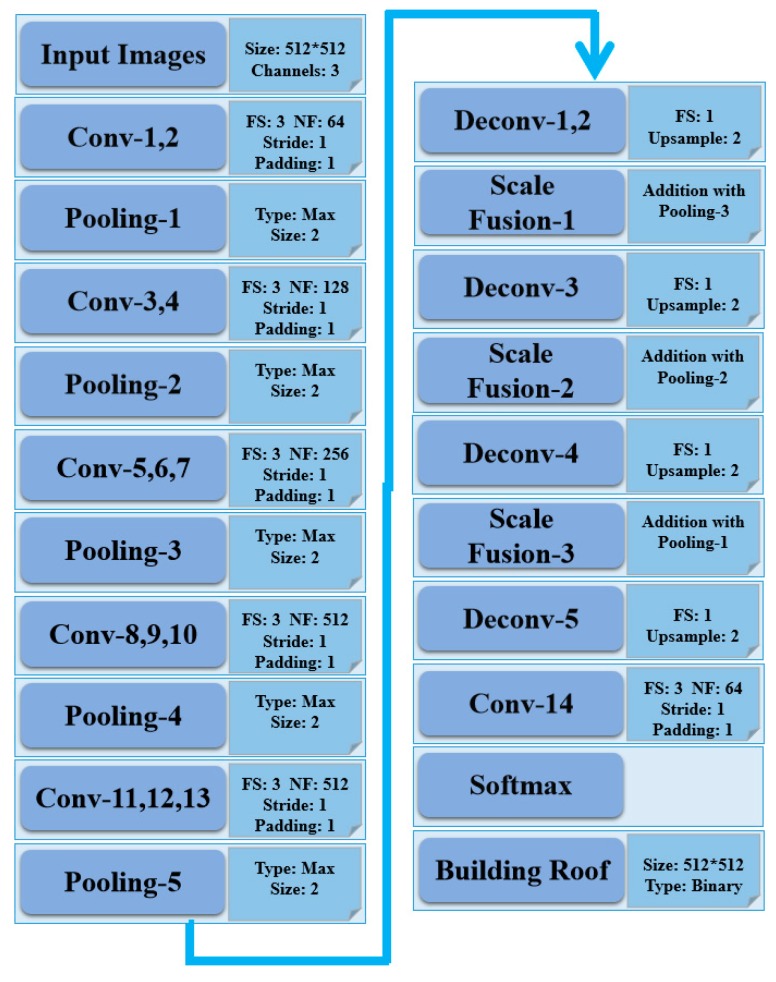
Architecture of the designed DCNN for building roof segmentation: Conv-N (N = 1–14) and Deconv-N (N = 1–5) denote the convolutional, deconvolutional fiters, respectively; Pooling-N (N = 1–5) are max pooling layers; Scale Fusion-N (N = 1–3) denotes the per-pixel addition layer for different features; FS, NF are filter size and number of filters.

There are many well-known CNN models for vision recognition tasks, e.g., AlexNet, VGG, GoogLeNet, RestNet. To develop an accurate model with high computational efficiency, the VGG-16 is selected as the basic DCNN network for building roof segmentation with GF2 VHR satellite imagery, according to Occam’s Razor. It provides promising results in different vision tasks with reasonable model size. The VGG-16 model is defined as a combination of several convolutional and pooling layers. However, the original VGG-16 model was designed to provide a summarized semantic information at the image level. Long et al., proposed a fully connected network (FCN) for dense prediction of images at pixel level, with the use of deconvolutional operation to upsample CNN layers [32], FCN provides an end-to-end framework for image segmentation at pixel level. Since the building roof mapping requires dense prediction, the deconvolutional layer is adopted to recover the image size for dense per-pixel prediction. Figure 2 illustrates the architecture of the DCNN model for the building roof segmentation with GF2 imagery, with both convolutional and deconvolutional layers. It is well addressed that the distribution drift of data, i.e., image Digital Number (DN) in this study, between layers in DCNN may significantly reduce the computational efficiency and segmentation accuracy. Batch normalization layers, as additional layers in fully convolutional DCNN, are therefore placed to perform feature normalization between layers. The fully convolutional network perform both feature extraction with spatial-spectral information of imagery and per-pixel class prediction.

#### 2.2.2. DCNN Model Training and Inference

To reduce the effect of varied illumination and atmosphere for the images acquired at different time over different areas, Equation (1) is employed to normalize the original image DN values at the scene level. This is expected to provide comparable training and test data sets. The normalized images are then clipped to sample images with a size of 512 × 512 pixels. In total 1460 sample images are selected, where 80% of the sample images are randomly selected for training, while the remaining 20% of the samples are selected for validation:

(1) $$DN_{T}=DN_{Ori}-\frac{\sum_{i=0}^{M•N}DN_{i}}{M•N}$$

where *DN_ori_* and *DN_T_* are the *DN* values of original and transformed images, respectively, and *M,N* are the image height and width. The DCNN model is implemented upon the open source deep learning package developed by Google, i.e., Tensorflow. High performance workstation with GPUs, i.e., NVIDIA TitanX, is employed to perform the DCNN model training and inference, and the batch size in the training is 8. The dropout rate of 0.5 is set at training stage to prevent the DCNN from over-fitting.

#### 2.2.3. Post-Processing with Conditional Random Field (CRF) 

It is well established that the convolutional and pooling operations in image segmentation can smooth the boundaries of objects [32]. To refine the segmentation results, CRF is employed to regularize the building segmentation. As a probabilistic graphical model, CRF has been widely applied in image analysis. It provides a solution for image analysis by connecting pixels with neighbors using a graph, the graph consists of nodes, which are single image pixels. The nodes, together with edges of the graph, are utilized to characterize spatial-spectral relationship. The posterior probability of the model can be characterized by the Gibbs distribution:

(2) $$\begin{array}{l} P(Y|X)=\frac{1}{Z(X)}\overset{-}{P}(Y,X)\\ \bar{P}(Y,X)=exp(\sum_{i}w_{i}*f_{i}(Y,X))\\ Z(X)=\sum_{Y}\exp(\sum_{i}w_{i}*f_{i}(Y,X)) \end{array}$$

where P(*Y|X*) is the normalized conditional probability of the event *X* and *Y*, which is characterized by a Gibbs distribution. P(*Y*,*X*) is the joint distribution probability, which is defined as an exponential function of weighted factors, and the factors are customized model for specific problems. Reference [31] proposed a fully connected CRF model for image segmentation upon pixel level, the joint distribution probability is parameterized with the summary of unary and pairwise potentials (Equation (3)):

(3) $$E(x)=\sum_{i}\psi_{u}(x_{i})+\sum_{i<j}\psi_{p}(x_{i},x_{j})$$

where *E(x)* is the Gibbs energy, $\underset{i}{\sum }\psi_{u}\left(x_{i}\right)$ is the unary term, which is computed independently for each pixel. $\underset{i<j}{\sum }\psi_{p}\left(x_{i},x_{j}\right)$ denotes the pairwise potentials, which are designed for characterizing the relationship between pixels and their neighbors. Detailed definitions of the unary and pairwise potentials can be found in [33]. With the mean field approximation algorithm, the maximization of the posterior probability of Gibbs distribution is calculated for inference of the model. In this study, the fully connected CRF model was employed for building roof map segmentation.

## 3. Results and Discussion

### 3.1. Training of the DCNN Model

**Figure 3:**
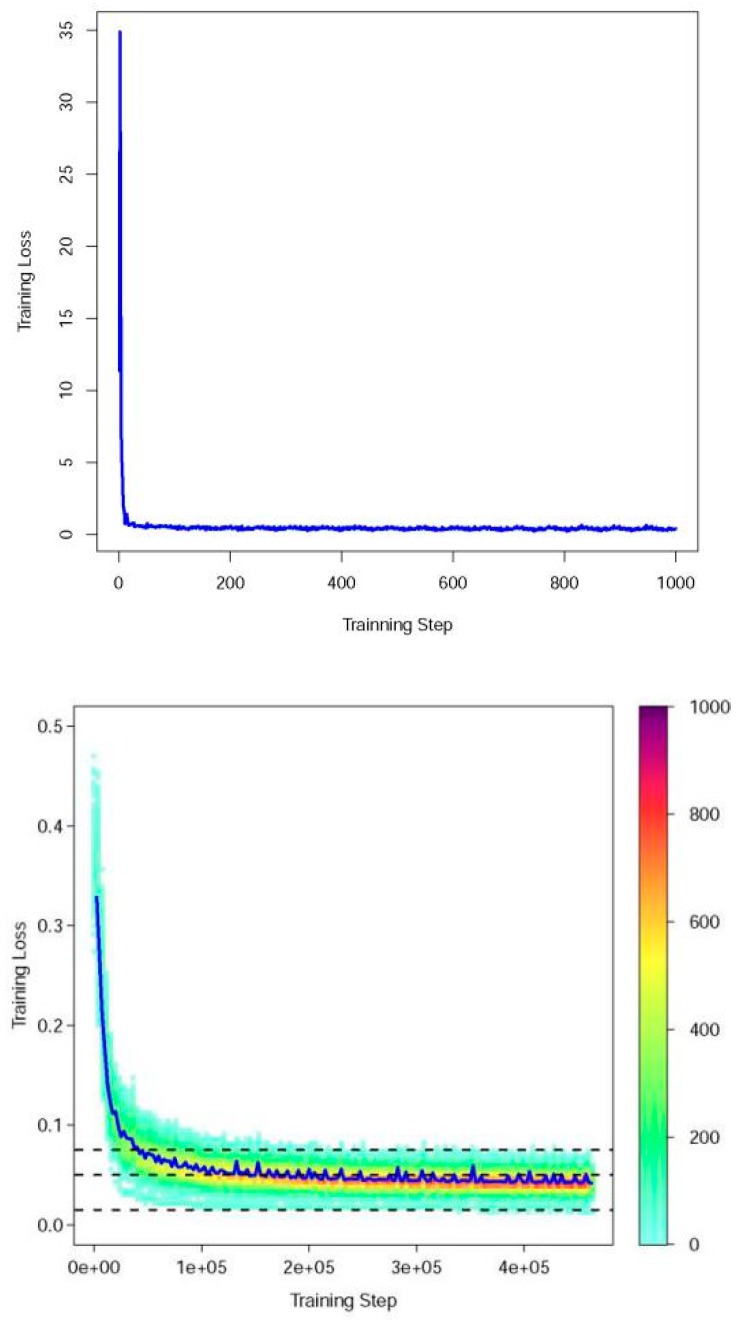
The change of loss value in model training stage: *X* axis and *Y* axis denote the training steps and the training loss, respectively; the upper figure shows the process with training step of 1–1000. The bottom figure shows the entire training process; the color of the bottom figure denotes the density of steps, while the dashed lines are training bounds with loss values of 0.075, 0.005, 0.015, and the blue line is the moving average of training losses with window size of 1000.

The batch size for the training is 8, with 3000 epochs for the optimization of the DCNN model. Figure 3 shows the change of loss in the training step, it suggest that the loss gradually decreased during the training stage. The moving average loss is calculated with moving window size of 100, while the maximum value of moving average error approximately is 1.2, the minimum value of moving average error is 0.05, and it does not have significant change in the end of training, that means the DCNN model converges to moving average error of 0.05.

### 3.2. Qualitative Assessment

**Figure 4:**
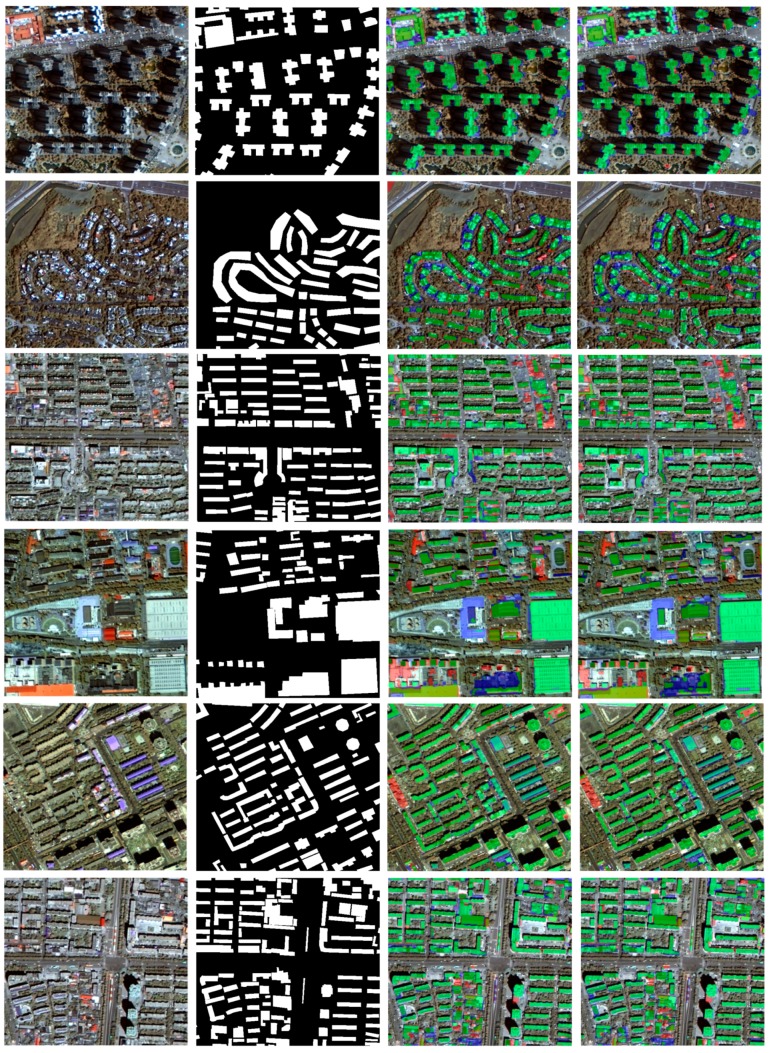
Visual comparison of the building roof segmentation results in different urban environment: for each row, images from left to right are true color composite GF2 PMS imagery (512 Pixel × 512 Pixel), manual delineation of building roof, DCNN segmentation results and CRF-refined building roof segmentation, respectively; for the images with segmentation results, green mask is True Positive (TP, building roof pixel was correctly classified), blue mask is False Negative (FN, building roof pixel was classified as non-building roof) and red mask is False Positive (FP, non-building roof pixel was classified as building roof).

Basically, the semantic segmentation of building roof is a binary classification, i.e., image pixels are labeled as either building roofs or non-building roofs. With the DCNN model trained by the interactive optimization, the segmentation of building roofs, i.e., the prediction of per-pixel label, is performed with the trained DCNN model. CRF is also employed to refine the segmentation results in this study. Figure 4 illustrates the building roof segmentation results with fused GF2 PMS imagery over the different study areas. Dense urban environments with business buildings, dense urban environments with apartments, low density environments with apartments, urban environments with single family houses and sparse urban environments, are selected for qualitative assessment. Colorized labels which indicate the comparison between prediction and ground truth are applied for visualizing the segmentation results in details. Visual inspection upon the segmentation results suggests that the DCNN could generate a promising building roof map over different urban environments. From the building roof map with colorized accuracy indicators, we can conclude that many individual buildings are segmented as connected image objects. While CRF is introduced as an efficient solution for optimizing the segmentation results, it doesn’t significantly change the building roof map. It is also observed that the segmented building roofs have smooth boundaries, even after the regularization operations with CRF. 

### 3.3. Quantitative Assessment

It is well known that quantitative assessment metrics are of paramount importance for land surface mapping. In the remote sensing community, the metrics were designed from the perspective of accuracy assessment of thematic maps at pixel level [34,35]. The DCNN model predicts building roof by labeling binary patches, unlike the pixel-wise classification, the accuracy of the semantic segmentation need to be assessed at both pixel level and patch level. Firstly, the per-pixel level accuracy is characterized by overall accuracy (OA), it is defined by Equation (4):

(4) $$OA=\frac{N_{TP}+N_{TN}}{N_{TP}+N_{TN}+N_{FP}+N_{FN}}$$

where *N_TP_*, *N_FN_* are the numbers of pixels which are correctly labeled, while *N_TN_*, *N_FP_* are the numbers of pixels which are incorrectly labeled. The mean overall accuracy is averaged with OA values of all test images. The mean intersection-over-union (mIOU) is applied to characterize the accuracy at segment level, and mIOU is calculated using Equation (5):

(5) $$\begin{array}{l} IOU=\frac{N_{GT}\cap N_{\mathrm{DR}}}{N_{GT}\cup N_{\mathrm{DR}}}\\ mIOU=\frac{\sum_{i=0}^{k}IOU_{i}}{k} \end{array}$$

where *N_GT_* is the total number of pixels of the ground truth, i.e., manually delineated building roofs. *N_DR_* is the total number of pixels of the corresponding building roofs detected by the DCNN model, and *k* is the total number of segmentation patches. The quantitative assessment of segmentation results are given in Table 3 and Table 4. The OA values of two results, i.e., DCNN segmented and CRF-refined building roofs are 94.67% and 94.69%, respectively. This suggests that the DCNN model worked very well for building roof segmentation using the Chinese GF2 imagery, and the CRF could improve the segmentation results. It is also suggests that the CRF could refine the results. However, the CRF-based refinement didn’t produce any significant changes to the original segmentation.

## 4. Conclusions

This paper presents an approach for semantic segmentation of building roofs in dense and diverse urban environment with Chinese GF2 PMS images. A fully connected DCNN is introduced to carry out the feature extraction as well as pixel labeling. Experiments are conducted upon VHR images acquired by the PMS on board the Chinese GF2 satellite. The DCNN could extract features in both the spectral and spatial domains, and the features are fused together for dense prediction at a pixel level. The quantitative assessment of the semantic segmentation results suggest that the fully connected DCNN achieved high accuracy building roof maps. However, the convolutional operation performs feature extraction with moving window filters, and the pooling operation aggregates neighboring pixels with summation, so both operations would smooth images and eventually change the boundaries of buildings. Future work on the algorithm improvement would focus on the fusion of low level geometrical features, e.g., multi-scale feature fusion with dilated convolutional filter, image transformation for building edge enhancement. 

It is demonstrated that the generation of DCNN models relies on a large volume of positive and negative samples, and the collection of massive numbers of building roof samples is not only costly, but it is also difficult to cover all scenarios in the practice of inference, especially in dense and diverse urban environments spread over large areas. Currently, there are some efforts being conducting to collect building roofs globally over different areas, e.g., SpaceNet, hosted by DigitalGlobe on Amazon Web Service (AWS), and the Volunteering Geographical Inventory (VGI) based approach is also being investigated for sample collection, which would be an alternative for large scale sample collection in future work.

In summary, the DCNN model performs well on semantic segmentation of building roofs in dense urban environments using GF2 VHR images. With the advances in high performance computers and the low cost GF2 imagery, it could be a promising method for operational building roof mapping over large areas with different building types and distribution patterns, and it offers an affordable data sources for both urban planning and sustainable development assessment.

## Figures and Tables

**Table 1:** Configuration of PMS.

Sensor	Spatial Resolution (m)	Spectral Bands (µm)
PMS-panromatic	0.8	0.45–0.90
PMS-multispectral	3.2	0.45–0.52; 0.52–0.59; 0.63–0.69; 0.77–0.89

**Table 2:** Area and acquisition date of images.

City	Region of China	Acquisition Date
Beijing	North	20160827
Shenyang	Northeast	20160612
Chengdu	Southwest	20160711
Guangzhou	South	20160723
Wuhan	Central China	20160901
Shanghai	Southeast	20160602
Urumqi	Northwest	20160625

**Table 3:** Overall accuracy and mIOU of the segmentation results.

Methods	OA	mIOU
DCNN	94.67%	0.83
DCNN-CRF	94.69%	0.83

**Table 4:** Confusion matrices for the segmentation results.

	Ground Truth	Building (DCNN)	NotBuilding (DCNN)	Building (CRF)	NotBuilding (CRF)
Segmentation					
		9968092	1902330	9447704	1399396
NotBuilding		2158790	62516836	2679178	63019770
